# Recent advances and developments in COVID‐19 in the context of allergic diseases

**DOI:** 10.1002/clt2.12065

**Published:** 2021-09-21

**Authors:** Mei Ding, Xiang Dong, Yuan‐li Sun, Milena Sokolowska, Mübeccel Akdis, Willem van de Veen, Ahmet Kursat Azkur, Dilek Azkur, Cezmi A. Akdis, Ya‐dong Gao

**Affiliations:** ^1^ Department of Allergology Zhongnan Hospital of Wuhan University Wuhan China; ^2^ Hubei Province Key Laboratory of Allergy and Immunology Wuhan University Wuhan China; ^3^ Swiss Institute of Allergy and Asthma Research (SIAF) University of Zurich Davos Switzerland; ^4^ Christine Kühne ‐ Center for Allergy Research and Education (CK‐CARE) Davos Switzerland; ^5^ Department of Virology Faculty of Veterinary Medicine University of Kirikkale Kirikkale Turkey; ^6^ Division of Pediatric Allergy and Immunology Department of Pediatrics Faculty of Medicine University of Kirikkale Kirikkale Turkey

**Keywords:** allergy, COVID‐19, mechanism, treatment, vaccination, Schlüsselwörter: Allergie, COVID‐19, Impfung, Mechanismen, Therapie

## Abstract

**Background:**

Since the first reports of coronavirus disease 2019 (COVID‐19) in Wuhan, China, in December 2019, there have been 198 million confirmed cases worldwide as of August 2021. The scientific community has joined efforts to gain knowledge of the newly emerged virus named severe acute respiratory syndrome coronavirus 2 (SARS‐CoV‐2), the immunopathological mechanisms leading to COVID‐19, and its significance for patients with allergies and asthma.

**Methods:**

Based on the current literature, recent advances and developments in COVID‐19 in the context of allergic diseases were reviewed.

**Results and Conclusions:**

In this review, we discuss the prevalence of COVID‐19 in subjects with asthma, attacks of hereditary angioedema, and other allergic diseases during COVID‐19. Underlying mechanisms suggest a protective role of allergy in COVID‐19, involving eosinophilia, SARS‐CoV‐2 receptors expression, interferon responses, and other immunological events, but further studies are needed to fully understand those associations. There has been significant progress in disease evaluation and management of COVID‐19, and allergy care should continue during the COVID‐19 pandemic. The European Academy of Allergy & Clinical Immunology (EAACI) launched a series of statements and position papers providing recommendations on the organization of the allergy clinic, handling of allergen immunotherapy, asthma, drug hypersensitivity, allergic rhinitis, and other allergic diseases. Treatment of allergies using biologics during the COVID‐19 pandemic has also been discussed. Allergic reactions to the COVID‐19 vaccines, including severe anaphylaxis, have been reported. Vaccination is a prophylactic strategy that can lead to a significant reduction in the mortality and morbidity associated with SARS‐CoV‐2 infection, and in this review, we discuss the proposed culprit components causing rare adverse reactions and recommendations to mitigate the risk of anaphylactic events during the administration of the vaccines.

## 1 PREVALENCE OF ALLERGY AND ASTHMA IN COVID‐19 PATIENTS

As the severe acute respiratory syndrome coronavirus 2 (SARS‐CoV‐2) primarily affects the respiratory tract, a preliminary hypothesis based on knowledge from common airway viruses proposed that asthma and other respiratory comorbidities might aggravate susceptibility to SARS‐CoV‐2 infection and lead to a more severe clinical outcome.^1^ However, early data from Wuhan reported a lower prevalence of asthma among COVID‐19 cases.^2^ Similar findings were observed in Italy, Brazil, and Russia,^3^, ^4^, ^5^, ^6^ even in severe asthma patients.^7^, ^8^ Other studies also suggested that asthma was not associated with delayed viral clearance,^9^ poor clinical outcome,^4^, ^10^, ^11^, ^12^ and mortality rate.^8^ Moreover, allergic status in children did not increase COVID‐19 incidence and its severity.^13^, ^14^ In contrast, published data in the United States and United Kingdom (UK) indicated an increased prevalence of COVID‐19 in patients with asthma.^15^, ^16^, ^17^ Severe asthma treated with a high dose of inhaled corticosteroid (ICS) + long‐acting beta 2‐agonist (LABA) presented a higher Intensive Care Unit admission rate due to COVID‐19.^18^, ^19^ Moreover, epidemiologic data from Korean disease Control and Prevention showed asthma was associated with an increased risk of mortality and worse clinical outcomes of COVID‐19.^20^ Skevaki et al. also suggested a complex relationship between prevalence and severity of COVID‐19 and allergy/asthma by reviewing more comprehensive epidemiologic data from different countries.^21^, ^22^

**FIGURE 1:**
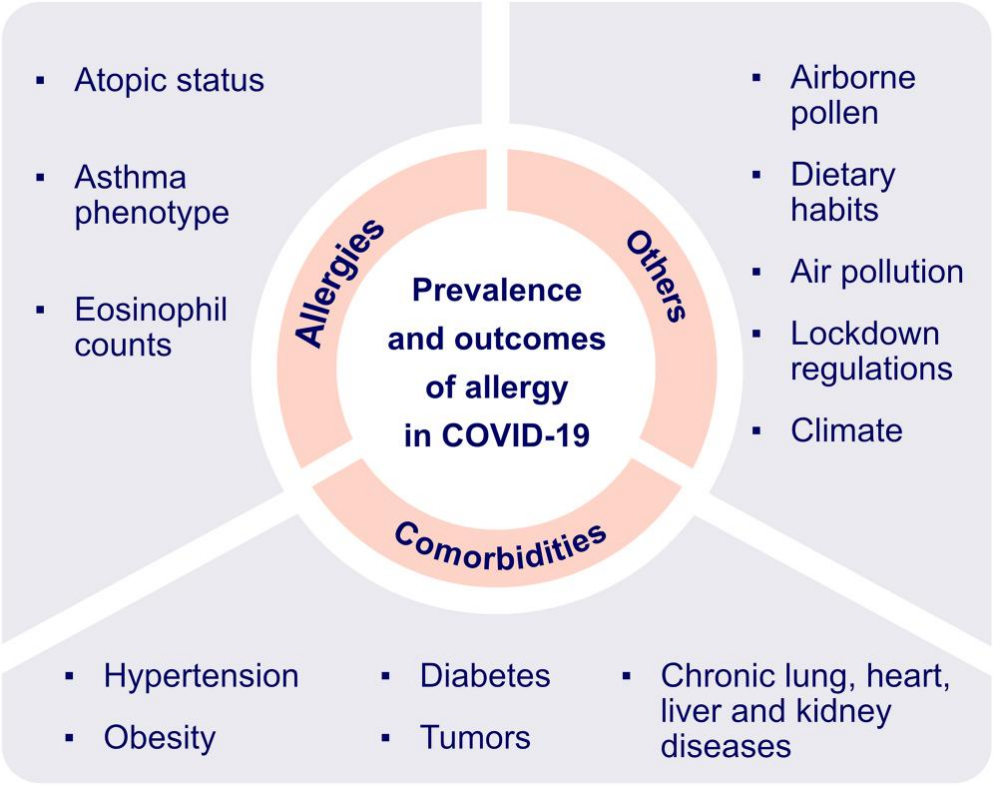
Potential factors associated with the prevalence and outcome of allergy in COVID‐19 patients. Atopic status is suggested to be associated with lower risk of SARS‐CoV‐2 infection. Asthma phenotype is found to be a strong determinant of disease severity in COVID‐19 with preexisting asthma. Lower eosinophil count is considered as predictive biomarker of severe COVID‐19. Airborne pollen concentration, dietary habits, lockdown regulations, climate and comorbidities might be responsible for inconsistent findings of prevalence of allergy in COVID‐19 patients

There are multiple factors that can account for these inconsistent findings including atopic status,^23^ asthma phenotype,^24^, ^25^ eosinophil counts,^24^ lockdown regulations,^12^, ^26^ dietary habits,^27^, ^28^, ^29^, ^30^, ^31^ airborne pollen concentrations,^32^, ^33^ air pollution,^34^ climate,^35^ and comorbidities^12^ (Figure 1). Respiratory atopy was suggested to have a protective role against severe lung disease in COVID‐19 patients with viral pneumonia.^23^ However, in contrast to patients with concomitant allergic rhinitis and asthma, allergic rhinitis alone was not regarded as a comorbidity that could modify susceptibility to SARS‐CoV‐2 infection as there was no significant difference in *ACE2* gene expression between allergic rhinitis subjects and controls.^36^

The asthma phenotype was found to be a strong determinant of disease severity. In a study from Stanford University, allergic asthma was found to mitigate the risk of hospitalization for COVID‐19 compared to patients with non‐allergic asthma (OR, 0.52).^24^ In addition, patients with non‐allergic asthma were more susceptible to SARS‐CoV‐2 infection and unfavorable clinical outcomes than patients with allergic asthma in a South Korean study.^25^ Lower eosinophil counts were a predictive biomarker of severe disease progression independent of asthma phenotype.^24^

Intriguingly, the prevalence of allergic diseases showed heterogeneous patterns during the COVID‐19 pandemic. Childhood asthma presented better outcomes with fewer asthma exacerbations leading to a reduced number of emergency visits and hospitalizations. Moreover, 66% of pediatric asthma patients had improved asthma control as measured by the improved forced expiratory volume in one second (FEV1) and the peak expiratory flow (PEF).^37^ In contrast, the number of hereditary angioedema attacks was notably increased due to restriction measures‐related anxiety, depression, stress and fear of COVID‐19.^38^, ^39^

A survey based on 14 member countries commissioned by the Asia Pacific Association of Allergy Asthma and Clinical Immunology (APAAACI) indicated an increased prevalence of allergic diseases among healthcare workers during the COVID‐19 pandemic, for example, ocular and airway allergy with extended use of surgical masks and eye protection; skin allergy due to prolonged use of gloves, protective equipment and frequent handwashing.^40^

Systemic allergic reactions during the pandemic seemed to be drastically reduced as indicated by the number of patients attending an Emergency Department Unit in the UK, from 62 in the first half of pre‐pandemic 2019 to 10 in the same period of 2020. The clinical manifestations presented before the pandemic by 52% of patients were classified as mild, according to the Brown grading system, whilst during the pandemic the majority of patients (80%) experienced moderate systemic allergic reactions.^41^

## 2 UNDERLYING MECHANISMS SUGGESTING A POTENTIAL PROTECTIVE ROLE OF ALLERGY IN COVID‐19

Allergy or atopy is characterized by a type 2 (T2) immune response against external antigens in individuals, in which genetic predisposition plays a major role.^42^ Mendelian randomization analysis of 136 uncorrelated single nucleotide polymorphisms with a broad allergic disease phenotype demonstrated a positive association between genetic predisposition to any allergic disease and lower risk of SARS‐CoV‐2 infection.^43^ ACE2 is the major receptor for SARS‐CoV‐2 entry into the cells. *ACE2* expression was reduced in differentiated airway epithelial cells treated with interleukin‐13 (IL‐13) or after exposure to cat allergen. Its expression was negatively correlated with allergic sensitization in nasal epithelium and Immunoglobulin E (IgE), IL‐13, fractional exhaled nitric oxide (FeNO) and other type 2 signatures. Similarly, transcriptomic data analysis suggested decreased *ACE2* expression in the nasal epithelium of children with allergic asthma and allergic sensitization or with asthma and/or allergic rhinitis.^44^, ^45^ The transmembrane serine protease 2 (TMPRSS2) cleaves the viral spike protein and regulates the interferon (IFN) response together with ACE2.^46^ Increased *TMPRSS2* expression was found in ex vivo airway epithelial cells in respiratory allergic subjects and was positively correlated with type 2 cytokines.^45^ T2 inflammation could also induce its expression in the metaplastic mucus secretory cells via IL‐13 signaling.^46^ Further studies were still needed to elucidate what atopy and asthma might render in the state of SARS‐CoV‐2 infection.

*ACE2* gene expression was downregulated in the airway epithelial cells, including in the nasal polys, and the olfactory neuroepithelium. Chronic rhinosinusitis with nasal polyps (CRSwNP) was characterized by a type 2 immune signature with increased eosinophil counts in the olfactory mucosa. Moreover, ACE2 expression was negatively correlated with the number of eosinophils.^47^ Pre‐existing eosinophilia was found to have a protective role as observed by a reduction in hospitalization with SARS‐CoV‐2 infection.^48^ In contrast, eosinopenia was predictive of poor disease outcome^49^ and was usually found in deceased COVID‐19 patients.^50^, ^51^, ^52^ A higher eosinophil count is a biomarker of allergic inflammation, and allergic subjects with eosinophilia were less susceptible to SARS‐CoV‐2 infection.^50^ Eosinophils were demonstrated to promote antiviral immunity in animal models.^53^ They are known to induce secretion of Th1 cytokines, generation of superoxide and nitric oxide (NO), and recruitment of CD8^+^ T cells against respiratory virus infection. Eosinophil‐derived enzymes have been proposed to neutralize the virus via a ribonuclease‐dependent mechanism.^54^ A lower eosinophil count was associated with CD8^+^ T cell depletion, which might be related to a Th17 inflammatory pattern in severe COVID‐19 patients.^55^, ^56^, ^57^ Further studies are warranted to confirm these findings.

**FIGURE 2:**
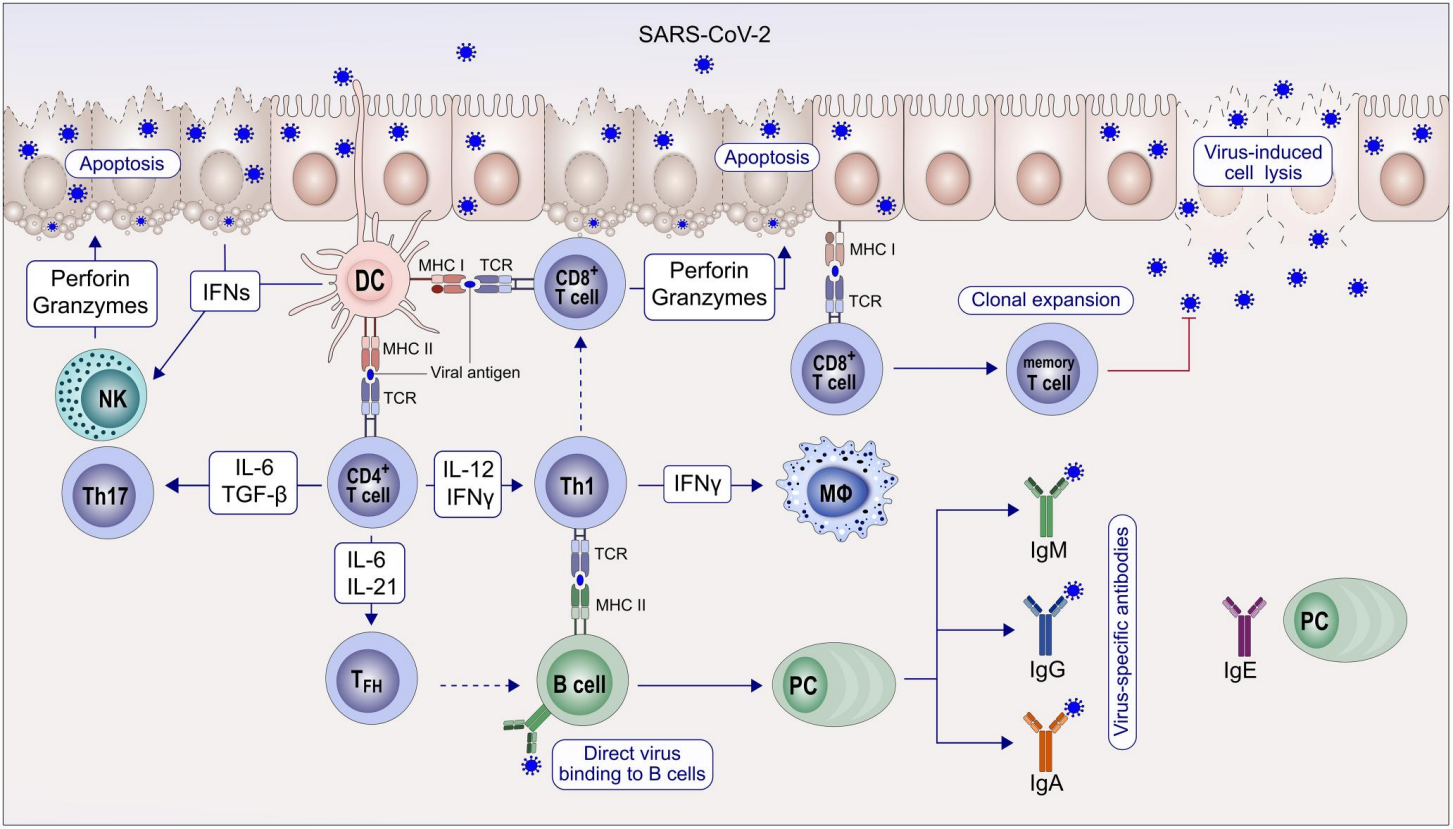
Immune responses to SARS‐CoV‐2. SARS‐CoV‐2 infection can cause epithelial cell lysis and directly destroy epithelium integrity. Subsequent to virus antigen presentation by dendritic cells, CD8+ T cells and natural killer cells induce cytotoxicity to infected epithelial cells and lead to apoptosis by releasing perforin and granzymes; CD4+ T cells differentiate into memory Th1, Th17 and memory T follicular helper (TFH). With the help of TFH, B cells develop into plasma cells (PC), contributing to virus‐specific antibodies production. In atopic subjects, IgE released by PC might play a negative role in the IFN‐α/β pathway regulation

Although type I and III interferons (IFN‐ α/β and ‐λ respectively) are essential to abrogate viral infection,^3^, ^58^, ^59^ excessive or prolonged type I IFN production promotes the release of proinflammatory chemokines that contribute to poor disease outcome by disrupting lung epithelial regeneration.^60^, ^61^, ^62^ In contrast, early or low type I IFN production has a protective effect against SARS‐CoV‐1 infection via regulation of monocyte/macrophage lung infiltration, vascular leakage, cytokine storm and T‐cell responses in SARS‐CoV‐1‐infected mice.^63^ Atopy has been reported to play a negative role in type I and III IFN production^64^, ^65^, ^66^, ^67^ by plasmacytoid dendritic cells (pDC) with impaired toll‐like receptor (TLR) expression and signaling cross‐linked by IgE and histamine H2 receptors^64^ (Figure 2). Since timing and robustness of type I IFN production is determinant for virus infection, more studies are furtherly demanded to explore the role of type I IFN in allergy and COVID‐19. Adaptive immune responses are also involved in allergy and modulate susceptibility to SARS‐CoV‐2 infection,^68^ for example, increased lymphocytes, notably T cells, were found in COVID‐19 patients with allergic comorbidities, suggesting milder SARS‐CoV‐2 infection in allergic patients.^69^

Various skin manifestations have been reported in SARS‐CoV‐2 positive patients,^70^, ^71^ there are emerging studies indicating a relationship between skin allergy and COVID‐19. Transcriptomic analysis showed that AD patients had a higher expression of *TMPRSS2* (encoding TMPRSS2), *PPIA* (encoding cyclophilin A), *SLC7A5* (encoding CD98), and other molecules involved in COVID‐19 pathophysiological mechanisms, both in lesional and non‐lesional skin.^72^ Moreover, based on the Proteomic Olink Proseek multiplex assay, a similar ACE2 expression pattern was found in healthy and AD subjects among adults and infant/toddlers, but elevated levels in the serum of adults with AD compared to infant/toddlers with AD. Cathepsin L/CTSL1 is a protein involved in the cleavage and priming of the SARS‐CoV‐1 spike protein and has been suggested to play a similar role in SARS‐CoV‐2 infection. Elevated levels of CTSL1 were found in the serum and were positively correlated with ACE2 protein expression.^73^

Treatment of allergic and non‐allergic asthma with ICS might also have an impact on the susceptibility to infection and COVID‐19 severity.

Even though the preliminary mechanistic data might suggest the protective role of type 2 inflammation against SARS‐CoV‐2 infection and COVID‐19 severity, further studies are urgently needed to evaluate these observations and to understand in detail these mechanisms in humans. This might also lead to the further development of new targets for pharmacological interventions against COVID‐19.

## 3 ALLERGY CARE DOES NOT STOP DURING COVID‐19

Although the COVID‐19 pandemic significantly impaired health care, management of allergic diseases was still ongoing by adhering to preventive measures. For example, there has been a shift of chronic urticaria consultation from face‐to‐face (decreased by 62%) towards telemedicine (increased by over 600%).^74^ Telecommunication tools have been implemented during the COVID‐19 pandemic,^75^, ^76^, ^77^ facilitating communication between doctors and patients whilst maintain social distancing. During the pandemic, mild‐to‐moderate or well‐controlled asthma patients were recommended to seek consultation online.^78^ Outpatient service was more appropriate for patients whose symptoms were not resolved or worsened with the escalation of medication.^78^, ^79^

Based on a European Academy of Allergy & Clinical Immunology (EAACI) survey, nearly half of newly referred asthma patients received face‐to‐face consultation with telemedicine follow‐ups. The majority of lung function tests were temporarily postponed. Initial asthma diagnosis and therapy were largely based only on clinical manifestations. All these factors contributed to the lower quality of healthcare during the lockdown. In general, the performance of lung function tests together with efficient remote monitoring presented the biggest challenges for asthma management in both children and adults.^80^

The small droplets (classically ≤5 microns) generated during pulmonary function tests contain SARS‐CoV‐2 and pose a significant risk of viral transmission in a healthcare setting.^81^, ^82^ Greening et al. quantified the mass of the aerosol particles emitted in different breathing maneuvers used to measure lung function. The mass of the exhaled particles was lowest during tidal breathing (TV), slow vital capacity following inspiration from functional residual capacity (sVC‐FRC) and forced expiratory volume (FEV). Coughing at total lung capacity generated the highest mass of exhaled particles. This data indicated that in the absence of coughing, spirometry did not pose a significant risk for viral transmission, which might be potentially beneficial for asthma diagnosis and follow‐up management.^82^

Telemedicine ensures the safety of both patients and healthcare professionals during the COVID‐19 pandemic, for example, quantitative measuring of olfactory dysfunction using psychophysical analyses were remotely performed.^83^, ^84^, ^85^ However, sputum induction, which is a widely used technique to evaluate airway inflammation and specially to classify the inflammatory phenotype of asthma can only be conducted in a hospital setting. As sputum induction generates aerosols it presents a high risk of viral transmission. In this case, the medical protocols were adapted to guarantee the safety of the patients and healthcare professionals, such as the use of personal protection equipment, and alternative sampling and processing procedures.^86^

EAACI launched a series of statements and position papers providing official recommendations for the treatment of drug hypersensitivity, allergic rhinitis, asthma, and other allergic diseases during the COVID‐19 pandemic (Table 1)^14^, ^87^, ^89^, ^90^, ^91^, ^92^. It also offered practical considerations on the organization of allergic clinics.^93^ In summary, atopic diseases should be treated following current guidelines even among patients at risk or with active SARS‐CoV‐2 infection. These guidelines should be continuously updated as we gain knowledge of this evolving coronavirus.

**TABLE 1:** The European Academy of Allergy & Clinical Immunology (EAACI) recommendations for the treatment of allergic diseases

Atopic disease	Key messages	References
Chronic rhinosinusitis (CRS)	Intranasal corticosteroids are recommended for CRS patients with SARS‐CoV‐2 infection, but systemic corticosteroids should be avoided. Surgery stays optional only with local complications or non‐responsive therapies.	[87]
Ocular allergy (OA)	Current EAACI recommendations for the management of OA^88^ remain the same during the COVID‐19 pandemic. Corticosteroids and immunomodulators should be used with caution especially for patients with active SARS‐CoV‐2 infection.	[89]
Drug hypersensitivity reactions (DHRs)	DHRs occurred rarely and most were nonimmediate cutaneous reactions. Disease‐related exanthems were the most characteristic differential diagnosis of DHRs.	[90]
Asthma	Inhaled corticosteroids or prescribed long‐term oral corticosteroids should continue. Spacers of large capacity are suggested to replace nebulization in patients with active SARS‐CoV‐2 infection.	[91]
Allergic rhinitis	Use of intranasal corticosteroids (including spray) should be continued.	[92]

## 4 ALLERGEN IMMUNOTHERAPY (AIT) AND BIOLOGICAL THERAPY FOR ALLERGY TREATMENT DURING COVID‐19

An APAAACI survey reported a decrease in AIT (46.1%) and immunosuppressive therapies (23.1%) in allergic patients during the COVID‐19 pandemic.^40^ Unfortunately, patients with non‐adherent subcutaneous immunotherapy (SCIT) for house dust mite allergy (≥2‐month delay) had higher median medication scores, visual analogue scale for quality of life, and total symptom scores.^94^ On the other hand, venom‐specific immunotherapy was safely administered in Spanish clinics following a strict sanitary protocol.^95^ Additionally, there was no reduced tolerability even among patients combined with early COVID‐19 symptoms and/or with positive SARS‐CoV‐2 results.^96^ Therefore, patients should be encouraged to adhere to treatment during the pandemic to ensure a successful outcome of immunotherapy.^94^, ^97^, ^98^

Biological therapeutics targeting type 2 inflammation pathways have been adopted in a wide range of allergic diseases.^99^, ^100^, ^101^ The safety of biologicals during the pandemic has come into question as these are known to interact with T2 cytokines and may interfere with eosinophil‐mediated antiviral activity. Reduced production of IgG and IgM and absence of IgA antibodies were observed in a severe asthmatic patient undergoing dupilumab treatment.^102^ Overall, a decreased (30.8%) use of biologicals in severe asthma was reported in an APAAACI survey.^40^ An Italian national registry of teledermatology services during the COVID‐19 pandemic showed that 1580 patients (86.3%) among 1831 patients continued therapy, in which 86.1% of patients continued dupilumab with a withdrawal rate of only 9.9%, albeit a higher interruption rate with systemic immunosuppressive agents. Discontinuation of treatment was mainly due to fear of SARS‐CoV‐2 infection from patients (39.9%), general practitioners (5.6%), and dermatologists (30.1%).^103^

Anti‐IgE antibody omalizumab has been reported to enhance the anti‐viral immune response by downregulating the high‐affinity IgE receptor on pDC.^44^, ^104^ Treatment of severe allergic asthma with omalizumab did not affect asthma control during symptomatic COVID‐19 disease.^8^, ^105^ The Italian Registry of Severe Asthma network showed no increased occurrence of SARS‐CoV‐2 infection among severe asthmatics treated with biologicals (omalizumab, mepolizumab, or benralizumab) compared to ICS + LABA alone.^7^ Moreover, compared to age and geography matched non‐asthmatic subjects, severe asthma patients with ongoing biological therapy did not show any increased incidence of SARS‐CoV‐2 infection.^106^

In summary, according to an evidence‐based EACCI statement, treatment with biologicals should be maintained in noninfected cases with continuous evaluation of atopic disease progression. In the case of active SARS‐CoV‐2 infection, biologicals need to be postponed until clinical resolution is established.^91^, ^107^, ^108^

## 5 VACCINATION OF COVID‐19 AND ALLERGY

Novel COVID‐19 vaccines are being developed as an indispensable prophylactic strategy to reduce the morbidity and mortality associated with COVID‐19.^109^, ^110^, ^111^, ^112^, ^113^, ^114^, ^115^ Classical signs of immediate allergic reactions have been reported within minutes of administration of SARS‐CoV‐2 vaccines in susceptible individuals, such as conjunctivitis, rhinorrhea, bronchoconstriction, generalized urticaria and/or angioedema. A few rare cases of anaphylaxis have been reported after vaccine administration.^116^, ^117^, ^118^, ^119^ The scientific community has made great efforts to understand the immunopathological mechanisms underlying allergic reactions to COVID‐19 vaccines and their culprit component.^120^, ^121^, ^122^, ^123^

Polyethylene glycol (PEG) has been identified as a potential trigger of allergic reactions. PEGs are found in many daily products and are an integral part of the micellar delivery system of the Pfizer‐BioNTech BNT162b2 and the Moderna mRNA‐1273 vaccines containing mRNA coding the spike protein of SARS‐CoV‐2.^120^, ^124^, ^125^ The pathological mechanisms underlying allergic reactions are not fully understood. Several studies suggest an IgE‐mediated reaction,^121^, ^126^, ^127^ whilst others have proposed a complement activation‐related pseudoallergy (CARPA) mediated by anaphylatoxins C3a and C5a together with anti‐PEG IgM and IgG antibodies induced by PEGyated nanobodies.^121^, ^127^, ^128^ Mast cell release might be associated with hyperreactivity to the vaccine in children with cutaneous mastocytosis.^127^, ^129^, ^130^ AstraZeneca AZD1222 vaccine contains polysorbate 80 which has been demonstrated to have cross‐reactivity with PEGs as they share the ether moiety –(CH_2_CH_2_O) _n_, which has been suspected as a causal excipient of hypersensitivity reaction to human papillomavirus vaccine.^131^, ^132^

There are also other potential culprit agents such as distearoylphosphatidylcholine (DSPC) in the Pfizer‐BioNTech and Moderna vaccines, formerly attributed to hypersensitivity to pollen. Trometamol is added as a buffer in the Moderna vaccine and there has been a case report of anaphylaxis to this excipient in an intravenously administered radiocontrast agent.^133^ Beta‐propiolactone (BPL), used to inactivate the virus, might induce an immune complex‐like reaction after vaccination,^121^ which might cause an adverse reaction after a booster dose injection of the rabies human diploid cell vaccine.^134^, ^135^ Similar immunological mechanisms might be implicated in the adverse reactions associated with two widely used COVID‐19 vaccines in China, BBIBP‐CorV (Sinopharm) and Sinovac‐CoronaVac (Sinovac Life Sciences), which also contain BPL to inactivate SARS‐CoV‐2.^121^

It is worth noting that allergic patients without a previous history to the vaccine components are not contraindicated for COVID‐19 vaccination.^136^ An allergological work‐up for patients with a possible risk of severe hypersensitivity reactions is recommended.^137^ The patients should be evaluated with skin tests for the vaccine and its excipients. Alternative vaccines that do not contain the suspected components could be considered in case of a positive skin result. In all cases, the vaccination should be followed with a minimum 15‐min observation. The vaccine should be administered in escalated doses for patients at a high risk of severe hypersensitivity reactions. An adrenaline (epinephrine) injector should be readily available to treat any anaphylactic event.^120^, ^133^, ^136^, ^138^

In conclusion, current evidence shows that allergy might play a protective role in COVID‐19 with the involvement of eosinophilia, SARS‐CoV‐2 related receptors expressions, and IFN responses, and other immunological events. Further research is warranted to improve our understanding of the underlying pathophysiological mechanisms. Allergy care has continued during the COVID‐19 pandemic and official recommendations from EAACI have been developed to inform healthcare professionals on the management and treatment of allergies during this time. Patients with a previous history of allergic reactions to a component of the COVID‐19 vaccines should be referred to an allergy clinic for a diagnostic workup.

There is a current need to improve our understanding of the underlying mechanisms involved in the vaccines adverse reactions and their culprit components to ensure their safety and public compliance.

## CONFLICT OF INTEREST

None of the authors has any conflicts of interest to declare.
